# Heat- and Ultrasound-Assisted Aqueous Extraction of Soluble Carbohydrates and Phenolics from Carob Kibbles of Variable Size and Source Material

**DOI:** 10.3390/foods9101364

**Published:** 2020-09-25

**Authors:** Chrystalla Antoniou, Angelos Kyratzis, Youssef Rouphael, Stelios Stylianou, Marios C. Kyriacou

**Affiliations:** 1 Agricultural Research Institute, P.O. Box 22016, 1516 Nicosia, Cyprus; chrystalla.antoniou@ari.gov.cy (C.A.); a.kyratzis@ari.gov.cy (A.K.); 2 Department of Agricultural Sciences, University of Naples Federico II, 80055 Portici, Italy; youssef.rouphael@unina.it; 3 Mellona, P.O. Box 54097-CY3720, 4151 Limassol, Cyprus; stelios@mellona.com.cy

**Keywords:** carob kibbles, carob juice, aqueous extraction, sugars, phenolics

## Abstract

Aqueous extraction of carob kibbles is the fundamental step in the production of carob juice and carob molasses. Improving the theoretical yield in sugars during organic solvent-free aqueous extraction is of prime interest to the food industry. Collateral extraction of phenolics, however, must be monitored as it influences the sensory and functional profile of carob juice. We presently examined the impact of source material, kibble size, temperature, and duration on the efficiency of extracting sugars and phenolics aqueously by conventional heat-assisted (HAE) and ultrasound-assisted (UAE) methods. Source material was the most influential factor determining the concentration of phenolics extracted by either method. Source material also influenced the relative proportions of sucrose, glucose, and fructose, which may impact the perceived sweetness of the juice. Kibble size (medium size M = 9–13 mm; powder size P = 1–4 mm) was more influential with UAE than HAE for both sugars and phenolics but was rendered less influential with prolonged UAE duration. Increasing HAE temperature (from 25 °C to 75 °C) favored the extraction of phenolics over sugars; however, prolonging extraction at 25 °C improved sugar yield without excessive yield in phenolics. Disproportionate extraction of phenolics over sugars limits the use of heat-assisted extraction to improve sugar yield in carob juice production and may shift the product’s sensory profile toward astringency. Prolonged extraction at near ambient temperature can, however, improve sugar yield, keeping collateral extraction of phenolics low. Ultrasound agitation constitutes an effective means of extracting sugars from powder-size kibbles. Industrial application of both methodologies depends on the targeted functional and sensory properties of carob juice.

## 1. Introduction

Carob is an evergreen species (*Ceratonia siliqua* L.) of the *Fabaceae* family. It is naturally self-propagated and cultivated in Cyprus as well as other countries of the Mediterranean basin, preferring mild and dry habitats [1]. In the last 5–10 years, carob cultivation has attracted renewed interest from growers driven by growing global demand for carob-based food products. According to the Food and Agriculture Organization statistics, the leader in the production of carob in 2018 was Portugal with 41,734 tons, followed by Italy, Morocco, Turkey, Greece, Cyprus, Algeria, Spain, and Lebanon [2]. The low-input cultivation practices required for carob production in combination with the rich bioactive constitution of its fruit renders carob a sustainable crop warranting further investigation. 

The carob fruit is a pod consisting of about 90% pulp and 10% seeds by weight at full maturity [3]. Carob seeds are in fact considered the most valuable part of the pods exploited industrially for the production of carob bean gum (locust bean gum, LBG), a widely used natural food additive [4]. Separation of the seeds from the pod by milling also delivers carob pulp kibbles as a low-cost by-product. Carob pulp kibbles have been used primarily as livestock feed, as cacao substitute in the confectionery and beverage industries, and as a regional culinary ingredient mostly in the Mediterranean basin [5,6]. In recent times, increasing attention has been given to products of carob pulp kibbles such as carob powder, fiber, juice, and molasses used by the food industry for developing a wide range of health-promoting or niche food products, including gluten-free ones [1,6]. Many studies have demonstrated the health-promoting effects of these products, notably their contribution to the prevention of colon cancer and hepatocellular carcinoma, wide antioxidant properties, reduction of diarrheal symptoms, and lowering of Low Density Lipoprotein cholesterol, as well as antidiabetic effects [7,8,9,10].

Carob kibbles are typically characterized by high sugar content (48–56% d.w.), crude fiber (around 40% d.w.), protein (2–7% d.w.), ash (2–3% d.w.), total polyphenols (1.4–2.0% d.w.), and low fat content (0.5–1% d.w.) [1,7,11]. Aqueous extraction of carob kibbles is performed conventionally at ambient conditions to deliver a juice rich in water-soluble sugars and phenolics [12]. Carob juice is a natural energy drink, widely consumed in Egypt, shown to have comparable sensory properties to grape juice, but higher phenolic content [13]. Carob juice can blend with other fruit juices to increase the nutraceutical value of the product due to the antioxidant and antimicrobial properties of polyphenols present in the carob juice [13,14,15]. The extraction of juice high in sugars is the first step in the industrial production of molasses (carob syrup) which is a concentrate of carob juice (65–80 °Brix) produced by slow simmering, without the addition of sugars or other additives [16]. Molasses is a nutritious, energy-rich, and healthy product that can be directly consumed or used as a functional ingredient alternative to sugar in the food and pharmaceutical industries [16,17]. The high sugar content of molasses renders it suitable for flavoring yogurt [18], while it is also used as a nutrient medium for *Aspergillus niger*, *Lactobacillus casei*, and the yeast *Saccharomyces cerevisiae* to produce citric acid and fermentation-derived lactic acid and bioethanol [1].

Several studies have been conducted to characterize the chemical composition of carob kibbles and to investigate the nutritional value and beneficial properties of kibble products [1,7,19,20,21,22]. To the best of our knowledge, however, very few works have examined the efficiency of organic solvent-free and time-efficient methodologies in extracting sugar-rich natural juice from carob kibbles, with the outlook of being applied on an industrial scale [19,23]. While the principal interest in such methods lies mainly in their sugar extraction efficiency, their yield in phenolic compounds must also be monitored as they may influence the oxidative stability and potential nutraceutical value but also the sensory profile of the products [13,24]. In this respect, the present short study examined the compositional differences in terms of soluble carbohydrates and phenolics of carob juice produced using two aqueous and environmentally friendly methods easy to apply at industrial scale extraction: 1. aqueous extraction under gentle agitation (conventional method—HAE) and 2. ultrasonic-assisted extraction (UAE). The HAE is traditionally applied by the industry for producing carob molasses. UAE, on the other hand, was examined briefly as a potential means of accelerating the extraction process. With each methodology, variable extraction parameters were appraised, including source material, kibble size, extraction duration, and temperature, in order to assess their comparative effect on the efficiency of extracting sugars and phenolics.

## 2. Materials and Methods

### 2.1. Plant Material

Fully mature carob pods were harvested from two principal phenotypes of local carob (*Ceratonia siliqua* L.) landrace: Lefkaritiki (LF) and Mavroteratsia (MV). These two phenotypes produce carobs of slightly different morphology although they are genetically proximate [25]. Carobs of Mavroteratsia were collected from a unitary source at low altitude (15 m) in the coastal zone (Zygi), while carobs of Lefkaritiki were collected from an inland unitary source at higher altitude (510 m) (Lefkara). As phenotype is the result of genotype-environment interaction, for the purposes of this study, we use the term “source material” to identify the lower-altitude material of Mavroteratsia and the higher-altitude material of Lefkaritiki.

### 2.2. Carob Juice Extraction

Carob pods were washed under tap water to remove debris, rinsed with deionized water, and patted dry. Then the pods were coarsely ground in a Vita Prep 3 blender (Vita-Mix Corp., Cleveland, OH, USA) operated at low speed and deseeded. The carob pulp was further ground to deliver kibbles of medium size (M = 9–13 mm) and powder size (P = 1–4 mm). Both sizes were placed in 50 mL falcon tubes and covered in distilled water (c. kibbles ≈ 15.3 g/H_2_O ≈ 26.1 g) to obtain a kibbles/water mass ratio of near 0.6. Extraction was performed either by a conventional heat-assisted method (HAE) or an ultrasonic-assisted method (UA). The HAE extraction was performed by incubating the tubes in an OLS200 Grant Instruments water bath (Cambridgeshire, UK) under gentle agitation (80 rpm) in a range of temperatures (25, 50, 75 °C) and durations (80, 160, 240, 320 min). The UA extraction was performed by placing the tubes in an S40H Elma ultrasonic water bath (Elma Schmidbauer GmbH, Singen, Germany) operated at 340 Watt power and 50/60 Hz frequency and thermostated at 40 °C for 30 or 60 min. Suspensions from both extraction methods were then strained through organza and centrifuged for 15 min at 19,341× *g*. Clear supernatants were used to determine nonstructural carbohydrates and total phenolics. All treatments were replicated three times.

### 2.3. Total Phenolics Content

The total phenolic content (TPC) of the aqueous extracts was determined according to the method of Singleton et al. (1999) [26] with slight modifications, previously described by Kyriacou et al. (2016) [27]. Quantification was performed on a Jasco V-550 UV-vis spectrophotometer (Jasco Corp., Tokyo, Japan) against linear calibration with external gallic acid standards over the range of 50–500 mg L^−1^, yielding a regression coefficient R^2^ > 0.99. The TPC of the extract was expressed in gallic acid equivalents (GAE) mg L^−1^.

### 2.4. Soluble Carbohydrates Content

For the analysis of water-soluble carbohydrates extract clarification was performed using Carrez Clarification Kit (Sigma-Aldrich, St. Louis, MO, USA). Separation and quantification of nonstructural carbohydrates (glucose, fructose, and sucrose) were accomplished by liquid chromatography on an Agilent HPLC system (Agilent Technologies, Santa Clara, CA, USA) equipped with a 1200 Series quaternary pump and a 1260 Series Refractive Index detector operated by Chem-Station software. Injection volume was 20 μL and separation was performed on a Waters 4.6 × 250 mm carbohydrate column (Waters, Milford, MA, USA) at 35 °C using an acetonitrile:water (82:18) mobile phase at a flow rate of 1.5 mL min^−1^. Quantification was performed against fructose, glucose, and sucrose calibrating curves established using six external standard concentrations (0.2–2.0 g 100 mL^−1^) with a coefficient of determination (R^2^) greater than 0.999 and expressed as g 100 mL^−1^.

### 2.5. Reagents and Standards

Gallic acid, fructose, Folin Ciocalteu’s Phenol reagent, and sodium carbonate were purchased from Sigma Aldrich (Steinheim, Germany). Glucose and methanol were purchased from Merck (Darmstadt, Germany). Sucrose was purchased from Fluka AG (Buchs, Switzerland) and acetonitrile was purchased form Supelco (Bellefonte, PA, USA).

### 2.6. Statistical Analysis

All analysis was performed using SPSS statistical package (IBM, SPSS, Chicago, IL, USA, ver. 25). Data were subjected to analysis of variance (ANOVA) and the percentage of total variance accounted for by the main effects and their interactions is presented. Mean comparisons were performed by Tukey’s b test. Two-tailed t test was used to compare mean values of treatments between the two extraction methodologies. Regression analysis on mean values was employed for profiling change in total phenolics content with extraction time at different temperatures for the two sources of material. Regression analysis was performed regardless of kibble size due to the very low percentage of variance explained by this factor.

## 3. Results and Discussion

Sugars and phenolics are the key components shaping the sensory and functional profile of carob juice. A moderate presence of phenolics may improve the stability and antioxidant capacity of the juice without imparting an astringent flavor at the expense of sweetness [14,19,27]. Carob pulp is high in sugars, chiefly sucrose (up to 52 g 100 g^−1^ d.w), fructose (1.8–12.5 g 100 g^−1^ d.w), and glucose (1.8–10.2 g 100 g^−1^ d.w), the relative content of which contributes differentially to perceived juice sweetness [7]. Cultivar and harvest maturity constitute factors that may substantially affect fruit sugar content and influence extraction efficiency [27,28]. In terms of phenolic constitution, carob pods contain mainly gallic acid, hydrolysable and condensed tannins, flavonol glycosides, and traces of isoflavonoids, the concentrations of which may also be influenced by cultivar and physiological stage of maturity at harvest [7,15,29,30].

Aside from the extraction of carob juice for consumption as beverage, the extraction of juice high in sugars also constitutes the fundamental step for industrial production of molasses (carob syrup), which is a concentrate of carob juice (65–80° Brix) produced by slow simmering, without the addition of sugars or other additives [16]. Several methodologies were previously examined concerning the extraction of sugars and phenolics from carob kibbles [22,23,30]; however, the parameters influencing the efficiency of aqueous extraction have received little attention despite the widespread application of aqueous extraction by the carob industry [23]. The efficiency of aqueously extracting sugars and phenolics from carob kibbles was presently assessed for heat-assisted and ultrasound-assisted extraction methodologies. The relative effect of key parameters (source material, temperature, duration, kibble size) on extraction efficiency was assessed through their relative contribution to the total variance of sugars and phenolics concentrations in the obtained carob juice (Table 1 and Table 2).

In the conventional heat-assisted method (HAE), variation in phenolics concentration was determined mainly by source material and temperature, while variation in total sugars concentration was not affected by temperature (Table 1). Variation in phenolics content foremost and sugars secondarily was observed in previous studies and attributed to genetic and environmental factors, harvest maturity, and postharvest storage [11,31,32,33]. Lefkaritiki delivered a juice higher in both sugars and phenolics than Mavroteratsia. The current results indicate that the source material can significantly affect the sugar and phenolics yield potential of aqueous extracts. This effect was more pronounced on phenolics than sugars, with Lefkaritiki yielding 3.6-fold higher phenolics concentration and 1.1-fold higher total sugars concentration than Mavroteratsia (Table 1). These phenotypes of the local carob landrace are genetically similar and bear limited differences in pod morphology. They were sourced, however, from different environments, with Lefkaritiki collected at 510 m altitude and Mavroteratsia near sea level. It has been previously established for arboricultural species as well as seasonal crops that phenolic content is highly modulated by abiotic stress conditions such as water stress, salinity, light intensity, and heat stress [34,35]. Given that carob constitutes an underutilized tree crop largely limited to cultivation in marginal lands of highly alkaline, calcareous, and infertile soils, where it subsists strictly under rainfed conditions, the impact of agro-environment on the phenolic content of the pod is unsurprising. Further research is therefore warranted to investigate the significance of growth environment on the actual pod content and ultimately juice extract concentrations of sugars and especially phenolics, variation in which can significantly influence the biological activity of carob kibble products [32].

**Figure 1:**
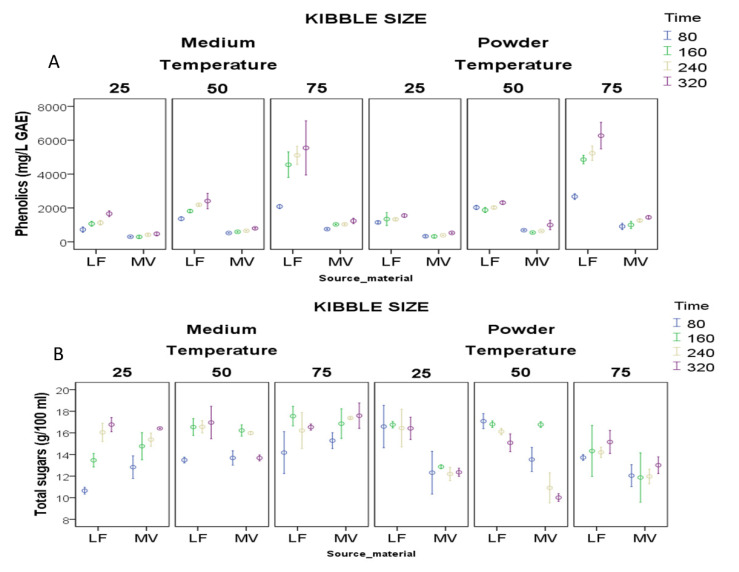
Interaction plots for total phenolics and sugars obtained by heat-assisted extraction (HAE; (**A**,**B**)) of different source materials (LF: Lefkaritiki; MV: Mavroteratsia) and kibble sizes (medium and powder). HAE extraction varied in time (80, 160, 240, 320 min) and temperature (25, 50, and 75 °C). Data points represent means of three replicates with standard error bars.

Kibble size had limited effect overall on the extraction of phenolics and moderate effect on sugars (Table 1). Similarly, a previous study demonstrated no effect of kibble size on the phytochemical composition of carob juice [13]. Significantly higher phenolics concentration was obtained with each incremental increase of extraction temperature and time (Table 1; Figure 1A). The most pronounced incremental increase in phenolics concentration was observed with both kibble sizes of Lefkaritiki when extraction temperature was raised to 75 °C and extraction time to 160 min (Figure 1A). Regression analysis highlighted the increase in phenolic concentration with progressive extraction time at each temperature (Supplementary Figure S1). The highest rate of increase in phenolic concentration over extraction time was observed for both source materials at 75 °C, yielding strong regressions (LF: R^2^ = 0.848, *p* < 0.001 and MV: R^2^ = 0.820, *p* = 0.002). Lefkaritiki was more responsive than Mavroteratsia, as shown by the slopes of the regressions (13.8 vs. 2.1, respectively). This observation is in accordance with previous findings that higher extraction temperatures yield higher phenolic concentrations [12,36]. Temperature and time effects on phenolic concentration may be partly explained by effective solubilization of condensed tannins abounding in carob pulp [19].

Extraction temperature overall had marginal effects on juice fructose and glucose concentrations and no effect on sucrose and total sugars concentrations (Table 1). Total sugar yield increased with time only for middle-sized kibbles extracted at 25 °C. It is worth noting that at 25 °C, the extraction of total sugars from middle-sized kibbles of Lefkaritiki was particularly increased when extraction was extended to 240 min, without eliciting an excessive concomitant extraction of phenolics (Figure 1B). According to Roseiro et al. (1991), the yield of carob carbohydrates aqueously extracted at ambient temperature (20 °C) increased with the extraction time, reaching a steady state after 6 h [12]. At higher than ambient temperatures, prolonging the extraction time had no apparent effect on sugars concentration while it increased the phenolics yield, thereby adding astringency to the juice owing in part to the presence of high molecular weight tannins [29,37,38]. High levels of tannins render carob juice more bitter in taste and metallic in aroma compared to grape juice [13]. On the other hand, phenolics and particularly hydrolysable tannins have gained substantial attention in the food and pharmaceutical industries as antioxidant and antimicrobial agents that improve the shelf life of a product [1,14]. Raising extraction temperature up to 75 °C for periods longer than 160 min has been presently shown effective in spiking the phenolic content of carob juice to be used as a stabilizing agent in juice blending.

**Figure 2:**
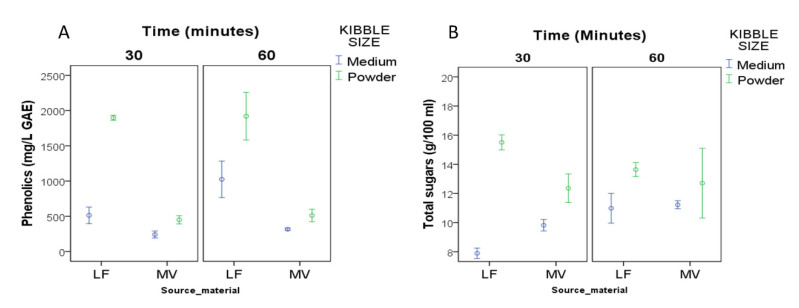
Interaction plots for total phenolics and sugars obtained by ultrasound-assisted extraction (UAE; (**A**,**B**)) of different source materials (LF: Lefkaritiki; MV: Mavroteratsia) and kibble sizes (medium and powder). UAE extraction varied in extraction time (30 and 60 min). Data points represent means of three replicates with standard error bars.

Kibble size was the most influential factor for the UAE extraction of sugars (Table 2). It also explained substantial variation in phenolics. The duration of sonication (30 vs. 60 min) had nonsignificant and minimal impact on the concentrations of sugars and phenolics in the sonicated aqueous extracts, respectively (Table 2; Figure 2A,B). However, the effect of kibble size on both sugars and phenolics extraction decreased when sonication time increased from 30 min to 60 min (Figure 2A,B ). As opposed to gentle agitation (HAE described above), sonication facilitated more efficient extraction from powdered than medium-sized kibbles (Figure 2A,B). This might be explained by the larger surface-to-volume ratio of powdered kibbles which were effectively kept in suspension by constant sonication, whereas in the case of gentle agitation, solvent circulation through powdered kibbles was impeded by sedimentation of the solid phase. This is an observation of practical significance for the carob industry since powder-sized kibbles are a major low-cost by-product of industrial carob milling, which inevitably ends up as animal fodder or source material for the production of carob juice and molasses.

Comparing the two extraction methodologies under similar temperature–time conditions by t-Test analysis, the HAE at 50 °C for 80 min (14.4 g 100 mL^−1^; 1150.3 mg L^−1^ GAE) demonstrated significantly higher total sugars and phenolics yield (P-sugars < 0.001, P-phenolics < 0.01) compared to UAE at 40 °C for 60 min (12.1 g 100 mL^−1^; 943.2 mg L^−1^ GAE). The lower phenolics concentration in the juice facilitated by UAE was therefore outweighed by the lower sugar yield obtained. Nonetheless, both methodologies can be applied potentially for industrial production of carob juice depending on the targeted functional and sensory properties of the extracted juice.

## 4. Conclusions

The results of this study provide an assessment of key parameters that affect the efficiency of aqueously extracting sugars and phenolics from carob kibbles. The source material was most influential in determining the concentration of phenolics in carob juice for both the heat-assisted and ultrasound-assisted extraction; however, the importance of the carob material’s environment of origin warrants further investigation in this respect. The source material also influenced the relative proportions of sucrose, glucose, and fructose in the juice, which bear an impact on the perceived sweetness of the latter. Of the extraction parameters examined, kibble size proved more influential in UAE than HAE for both sugars and phenolics by facilitating better suspension and preventing sedimentation of powder-sized particles. Kibble size was less influential as UAE duration increased. In the case of HAE, increasing extraction temperature favored the yield in phenolics over sugars, thus shifting the sensory profile of the juice toward astringency. However, prolonging the extraction time at 25 °C improved sugar yield without excessive extraction of phenolics.

## Figures and Tables

**Table 1:** Percentage of variance explained by main effects and mean comparisons for fructose (FRU), glucose (GLU), sucrose (SUC), total sugars, and phenolics obtained by conventional extraction (HAE) using two source materials (LF: Lefkaritiki; MV: Mavroteratsia) and two kibble sizes (M: medium-size kibbles; P: powder-size kibbles) in a range of extraction temperatures (25, 50, and 75 °C) and durations (80, 160, 240, 320 min). GAE: gallic acid equivalents. Interaction plots of main effects on sugars and phenolics are presented separately in Figure 1A,B.

Source of Variation	Fructose	Glucose	Sucrose	Total Sugars	Phenolics
	(g 100 mL^−1^)	(g 100 mL^−1^)	(g 100 mL^−1^)	(g 100 mL^−1^)	(mg L^−1^ GAE)
*Source material*	*Means*				
LF	2.6b	1.7b	11.3a	15.6a	2595a
MV	3.4a	2.5a	8.2b	14.0b	715b
*Kibble size*					
M	3.0a	2.1	10.4a	15.5a	1573b
P	2.9b	2.1	9.1b	14.1b	1737a
*Temperature*					
25	2.8b	2.1ab	9.7	14.5b	830c
50	3.1a	2.10a	9.8	15.0a	1341b
75	3.0a	2.0b	9.9	14.9ab	2844a
*Time*					
80	2.7b	1.8b	9.2c	13.8b	1127d
160	2.9b	1.9b	10.6a	15.4a	1608c
240	3.1a	2.2a	9.8b	15.0a	1840b
320	3.2a	2.3a	9.5bc	15.0a	2148a
	*Percentage of Variance*				
*Source materials*	42.5 ***	50.3 ***	39.9 ***	12.9 ***	37.4 ***
*Kibble size*	0.8 *	0	6.3 ***	9.6 ***	0.3 **
*Temperature*	4.0 ***	1.0 *	0.1	0.8	30.0 ***
*Time*	13.8 ***	9.6 ***	4.5 ***	7.8 ***	5.3 ***

* Significant effect at the 0.05 level; ** significant effect at the 0.01 level; *** significant effect at the 0.001 level. Data represent means of three biological replicates. Means followed by different lowercase letters within each column denote significant difference at *p* < 0.05.

**Table 2:** Percentage of variance explained by main effects and mean comparisons for fructose (FRU), glucose (GLU), sucrose (SUC), total sugars, and phenolics obtained by ultrasound-assisted extraction (UAE) using two source materials (LF: Lefkaritiki; MV: Mavroteratsia) and two kibble sizes (M: medium-size kibbles; P: powder-size kibbles) under two extraction times (30 and 60 min). Interaction plots of main effects on phenolics and sugars are presented separately in Figure 2A,B, respectively.

Source of Variation	Fructose	Glucose	Sucrose	Total Sugars	Phenolics
	(g 100 mL^−1^)	(g 100 mL^−1^)	(g 100 mL^−1^)	(g 100 mL^−1^)	(mg L^−1^ GAE)
*Source material*	*Means*				
LF	1.8b	1.4b	8.8a	12.0	1338.8a
MV	2.5a	1.9a	7.1b	11.5	379.9b
*Kibble size*					
M	1.9b	1.6	6.5b	10.0b	523.9b
P	2.5a	1.7	9.3a	13.6a	1194.9a
*Time*					
30	2.1	1.7	7.6b	11.4	775.7b
60	2.3	1.7	8.2a	12.1	943.1a
	*Percentage of Variance (%)*				
*Source material*	40.1 ***	52.0 ***	18.7 ***	1.1	53.8 ***
*Kibble size*	33.2 ***	4.6	48.2 ***	59.7 ***	26.3 ***
*Time*	2.3	0.0	2.1 *	2.6	1.6 **

* Significant effect at the 0.05 level; ** significant effect at the 0.01 level; *** significant effect at the 0.001 level. Data represent means of three biological replicates. Means followed by different lowercase letters within each column denote significant difference at *p* < 0.05.

## Supplementary Materials

The following are available online at https://www.mdpi.com/2304-8158/9/10/1364/s1, Figure S1: Regression of total phenolics concentration in carob juice with extraction time (60, 180, 240, and 320 min) using heat-assisted conventional method over a range of temperatures (25, 50, 75 °C) for the two source materials (LF: Lefkaritiki; MV: Mavroteratsia).
