# Supplementary material for: Heat- and Ultrasound-Assisted Aqueous Extraction of Soluble Carbohydrates and Phenolics from Carob Kibbles of Variable Size and Source Material
Source: Foods. 2020 Sep 25;9(10):1364. doi: 10.3390/foods9101364 (PMC7599798; doi:10.3390/foods9101364)
Supplement: Supplementary file 1 [file foods-09-01364-s001.pdf]

Supplementary Material:

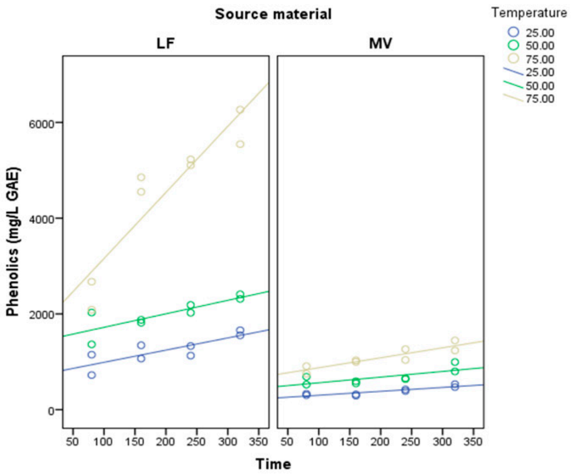

Model Summary and Parameter Estimates

Dependent Variable: Phenolics

Source material			Model Summary					Parameter Estimates	
			R Square	F	df1	df2	Sig.	Constant	b1
LF	25.00	Linear	.678	12.636	1	6	.012	734.433	2.547
	50.00	Linear	.673	12.330	1	6	.013	1437.842	2.821
	75.00	Linear	.848	33.357	1	6	.001	1777.625	13.806
MV	25.00	Linear	.808	25.190	1	6	.002	219.350	.815
	50.00	Linear	.549	7.291	1	6	.036	442.758	1.184
	75.00	Linear	.820	27.321	1	6	.002	666.383	2.087

The independent variable is Time.

Figure S1. Regression of total phenolics concentration in carob juice with extraction time (60, 180, 240, and 320 min) using heat-assisted conventional method over a range of temperatures (25, 50, 75 °C) for the two source materials (LF: Lefkaritiki; MV: Mavroteratsia). Data points represent means of three replicates.
